# Public Engagement with Climate Change and Health: A Global Literature Review

**DOI:** 10.1007/s10393-025-01756-4

**Published:** 2025-10-20

**Authors:** Sri Saahitya Uppalapati, Eryn Campbell, John Kotcher, Kathryn Thier, Patrick Ansah, Neha Gour, Edward Maibach

**Affiliations:** 1 Center for Climate Change Communication, George Mason University, Fairfax, VA 22030 USA; 2 Penn Center for Science, Sustainability, and the Media, University of Pennsylvania, Philadelphia, PA 19104 USA; 3 Department of Communication, Fairleigh Dickinson University, Teaneck, NJ 07666 USA

**Keywords:** climate change, public health, climate change communication, public perceptions

## Abstract

With the impacts of climate change on health becoming increasingly severe and far-reaching, effective communication to diverse audiences is more crucial than ever. This review analyzes 93 studies published between 2000 and 2023 on public understanding and responses to information about climate change and health. We synthesize research on public perceptions of climate change and health, responses to health-framed climate information, and information about climate and health risks and solutions, and the depolarizing potential of health messaging. Our findings suggest that conveying the health relevance of climate change holds significant potential for enhancing public engagement and building support for climate action. Additionally, we identify research gaps, particularly in understanding how different demographic audiences perceive health-related climate information and suggest directions for future studies. This synthesis of international research provides valuable insights into how different populations perceive and react to health-related climate information, highlighting the importance of targeted and effective communication strategies in addressing the climate crisis. The findings and summaries in this review can serve as valuable tools for evidence-based initiatives to address the critical issue of climate change and its profound implications for public health.

**Electronic supplementary material:**

The online version of this article (10.1007/s10393-025-01756-4) contains supplementary material, which is available to authorized users.

## Introduction

As climate change worsens, the consequences on human health and health systems are becoming increasingly common and severe (IPCC, 2022). Rising temperatures, extreme weather events, poor air quality, and other impacts of climate change pose significant threats to public health, exacerbating existing health disparities and straining healthcare infrastructure.

Developing effective communication strategies to make people aware of the link between climate change and health is critical to building public and political will for concerted action on climate change (Romanello et al., 2023). Effective communication can drive policy changes and inspire individual and collective actions that mitigate climate risks and promote health benefits (Howard et al., 2023; Maibach et al., 2023). To provide a comprehensive, global synthesis of the literature on public awareness of the connection between climate change and health, as well as evidence of effective communication strategies for discussing the health relevance of climate change, here we review research published from 2000 to 2023 in several languages, including English, Chinese, French, German, Italian, Japanese, Korean, Portuguese, and Spanish. Furthermore, we identify significant research gaps and opportunities for researchers and practitioners in the field of climate change and health.

## Methods

Our search strategy was tailored to identify relevant English and non-English publications. For English-language publications, we conducted an iterative search process. Our search spanned research articles published from January 2000 to July 2023, utilizing Google Scholar and key scholarly databases, including ScienceDirect, PubMed, and Web of Science. Given the extensive number of results, we sorted them by relevance and reviewed the first 15 pages from each database.

To broaden the scope and generalizability of our findings, we also included studies published in languages other than English. In collaboration with a research librarian, we identified several international databases such as SciELO, CNKI, Erudite, J-Stage, Korean Citation Index, and CyberLeninka, which feature research in Chinese, French, German, Italian, Japanese, Korean, Portuguese, Russian, and Spanish. We tailored search strings for each database, adjusting for those requiring more specific terminology. These databases allowed searches in English, which obviated the need for a prior translation of search terms into each respective language. In addition to the scholarly databases, we also used Google Scholar because of its capability to search for research published in non-English languages.

The initial screening of search results was performed by a single author who reviewed titles and abstracts. For those not available in English, DeepL, an online translation service, was used to translate them into English. If a study was deemed relevant from its title and abstract, at least two authors proceeded to a full-text review, again using DeepL for translation when necessary.

Furthermore, we actively sought relevant citations from gray literature and partner organizations such as the Global Climate and Health Alliance, the Australian Climate and Health Alliance, Health Canada, the Canadian Medical Association, and the WHO Civil Society Working Group on Climate Change. This outreach was conducted during working group meetings and through solicitation via email listservs.

## Findings

As part of a broader project (which also included a focus on health professionals and public officials), we identified 182 English-language and 13 non-English-language studies that explore the perspectives of the public, health professionals, and public officials on the health impacts of climate change and the evaluation of various communication strategies for these impacts and future risks.^1^ Of these, 93 studies (87 in English and 6 in non-English languages) focused on public audiences (see Supplementary materials for a list of the articles reviewed, the language in which they were published, their geographical scope, and information on the populations).

Below, we organize and synthesize the findings from these studies into four distinct themes: public perceptions of climate change and health; the effectiveness of health framing (as compared to other frames); public responses to information about health risks associated with climate change and the health benefits of climate solutions; and the potential of the health frame to depolarize the dialogue on climate change.

**Table 1:** provides an overview of our eligibility criteria for inclusion. Table S1 in the Supplementary materials overviews the search results and search strings used for English and non-English language research across various databases.

Criteria	Inclusion	Exclusion
Population	General Public	Professionals in specific fields (e.g., journalists, business leaders)
Concept	Articles focusing on the views of the public regarding the health implications of climate change; articles evaluating different strategies for effectively communicating about current and future health risks of climate change and the health benefits of action to address climate change	Articles about aspects of climate change and health are not specifically assessments of the views of the general public or assessments of effective strategies to communicate with the public; articles address climate change or environmental issues broadly and not climate change and health specifically
Time	Published from January 2000 to July 2023	Published before 2000 and after July 2023
Article type	Primary research and gray literature	Commentaries, media pieces, blogs, newspaper articles, and dissertations
Language	Chinese, English, French, German, Italian, Japanese, Korean, Portuguese, and Spanish	Languages not listed (excluding those specified)

### Public Perceptions of Climate Change and Health

The available evidence on public perceptions of the health relevance of climate change is limited, primarily due to small or unrepresentative sample sizes in studies that are often restricted to specific cities or subnational regions. We identified research conducted across various regions: Asia (17), Africa (10), North America (9), South America (4), Europe (including the UK; 6), the Caribbean (1), and two studies spanning multiple continents. Of these, only eleven studies utilized nationally representative samples—one in Asia, seven in North America, two in Europe, and one multinational.

### US, UK, and Canada

Findings from several studies conducted in the US suggest that many Americans believe that climate change and air pollution from burning fossil fuels pose a threat to human health (Akerlof et al., 2010; Akerlof & Maibach, 2015; Cutler et al., 2018; Hathaway and Maibach 2018; Kotcher et al., 2019a, 2020; Maibach et al., 2015; Madrigano et al., 2018; Roser-Renouf et al., 2021), although few possess specific knowledge of how climate change harms health and who is most likely to be harmed (Akerlof et al., 2010; Maibach et al., 2015). The earliest study—which reported nationally representative survey findings from the US, Canada, and Malta—found that a majority of Americans (as well as Canadians and Maltese) acknowledged that climate change poses significant health risks, but few were able to name specific climate and health risks in response to an open-ended question (Akerlof et al., 2010). This same pattern of findings was replicated in a subsequent study of Americans five years later, which also found that few could name specific groups of Americans whose health was most likely to be harmed by climate change (Maibach et al., 2015). Similarly, a later study revealed that while 73% of Americans were aware that exposure to air pollution from fossil fuel combustion harms health, only 55% could name even a single health consequence of such pollution (Kotcher et al., 2019a).

More recently, Americans appear to have gained an improved recognition of the health implications of climate change (Kotcher et al., 2020; Roser-Renouf et al., 2021). For example, in 2020, 54% of Americans expected the health risks from wildfire smoke in their community to increase over the next decade, which was up from only 26% in 2014 (Kotcher et al., 2020).

Americans' perceptions of the health consequences of climate change are strongly associated with their broader climate attitudes and beliefs (Cutler et al., 2018; Roser-Renouf et al., 2021). Between 2014 and 2020, the audience segments known as Global Warming’s Six Americas—ranging from the Alarmed (those who understand the threat of climate change and are very worried about it) to the Dismissive (those who think climate change is not real and are likely to believe it is a hoax)—learned about the links between climate change and health at vastly different rates. There was considerable learning among the Alarmed, Concerned, Cautious, and Disengaged segments, but little or no learning among the Doubtful and Dismissive segments (Roser-Renouf et al., 2021). Additionally, political ideology, age, race, and income have been shown to influence perceptions of heatwave risk, with white individuals, conservatives, and those with higher incomes less likely to perceive health risks of heatwaves (Cutler et al., 2018; Madrigano et al., 2018; Speiser & Hill, 2021; Stoutenborough et al., 2014). Furthermore, other factors such as social and health vulnerabilities also affect perceived climate-related health risks (Akerlof et al., 2015).

Canadians appear to be somewhat more aware than Americans of the health harms associated with climate change. Open-ended survey responses revealed that over half (58%) of Canadians could name one or more health impacts of climate change, most commonly impacts related to food security and agriculture, air quality, temperature-related morbidity and mortality, and extreme events (Casson et al., 2023). However, this level of awareness represented a slight decline from the 63% found in a similar Canadian survey conducted in 2008 (Environics Research Group, 2008). Thus, it appears that public awareness of the health impacts of climate change in Canada hasn’t grown over the past decade (Casson et al., 2023).

UK-based studies suggest that public perceptions vary depending on the specific climate impact and personal experience with climate effects. A representative survey of UK adults found widespread concern about the effects of climate change on UK residents’ health, particularly regarding air pollution (67%) and severe floods (68%; Harrison & Graham, 2022). Those who had experienced air pollution and flooding in the past year were twice as likely to say that local governments should prioritize addressing those impacts (Harrison & Graham, 2022). However, participants from qualitative interviews conducted in England said they were unsure if the UK was experiencing climate change and found it difficult to see the connection between climate change and health (Martin-Kerry et al., 2023).

### Other Countries & Regions

Several studies have found relatively high-risk perceptions related to the health impacts of climate change in regions and countries such as Bangladesh, China, Cyprus, Ethiopia, India, Hong Kong, Kenya, Tanzania, Thailand, Laos, Germany, Chile, Malaysia, South Africa, Vietnam, Nepal, Tibet, South Korea, Germany, Indonesia, Brazil, Thailand, and the Caribbean (Alfaro & Cortés, 2020; Bambrick et al., 2015; DeBono et al., 2012; Drewry et al., 2022; Fitchett & Swatton, 2020; Gao et al., 2020; Hossain et al., 2021; Kabir et al., 2016; Konstantinou et al. 2022; Leiserowitz et al., 2022; Li et al., 2019; Mayala et al., 2015; Mahata & Shekar, 2023; Ock et al., 2018; Rahman et al., 2014, 2021; Rodríguez et al., 2020; Sulistyawati et al., 2018; Toan et al., 2014; van Baal et al., 2023; van Wijk et al., 2020; Wang et al., 2022).

Only two studies, conducted in India (Leiserowitz et al., 2022) and Malta (Akerlof et al., 2010; DeBono et al., 2012) used nationally representative samples. In India, more than half of the participants said that global warming would cause "many more" disease epidemics (59%) and food shortages (49%). In Malta, nearly all respondents (89%) acknowledged that climate change could cause illness, with nearly two-thirds (63%) stating that it is happening now (Akerlof et al., 2010). Furthermore, the perception that climate change poses a risk to one’s health and general well-being was the strongest predictor of support for climate change mitigation policies and a willingness to take individual-level actions to mitigate climate change (DeBono et al., 2012).

The remaining studies used small or non-representative samples, making it challenging to draw conclusions; however, they provide valuable insights. Notably, some of the studies suggest that people perceive increasing health harms associated with changing climatic conditions in their area, such as droughts or changes in rainfall (de Moura Brito Júnior et al., 2023; Haque et al., 2012; Hossain et al., 2021; Kabir et al., 2016; Li et al., 2019; Mahata & Shekar, 2023; Toan et al., 2014; Torres-Slimming et al. 2021; Tripathi et al., 2021). For example, in Bangladesh, riverine island dwellers, who are particularly vulnerable to climate change due to geographic and socioeconomic factors, reported experiencing worsening health impacts during extreme weather events and natural disasters (Hossain et al., 2021).

Studies conducted in several African countries—including Tanzania, Nigeria, Ethiopia, Uganda, Kenya, and Senegal—show widespread awareness of the health risks of climate change and, in some cases, a deep understanding of the specific ways in which climate change may affect their health (Ajuang et al., 2016; Armah et al., 2015; Asekun-Olarinmoye et al., 2014; Boamah et al., 2017; Bambrick et al., 2015; Bryson et al., 2021; Mayala et al., 2015; Mbaye, 2015). For instance, in Uganda, Indigenous women participating in focus groups shared that long-term changes in seasonal patterns (i.e., “climate change”) are exacerbating seasonal food insecurity, which in turn affects maternal health during pregnancy and infant health (Bryson et al., 2021).

In contrast, few studies have found limited awareness of the links between climate change and health (Hussey & Arku, 2019; Petrescu-Mag et al., 2023; Pitpitunge, 2013). For example, Romanian Millennials and Generation Z participants had low awareness of the link between climate change and health, even though they noted changes in average summer and winter temperatures (Petrescu-Mag et al., 2023).

Two studies found that some vulnerable groups may not perceive themselves as at greater risk. Patients in alpine Swiss and German pulmonary rehabilitation programs did not report an increased vulnerability to climate change compared to healthy tourists, even though both groups expected greater future impacts (Götschke et al., 2017). Similarly, a recent survey of Chinese outdoor construction workers found low overall awareness of heat risks (Han et al., 2021).

Overall, studies indicate that many people are aware of the health impacts of climate change and extreme weather events. While public awareness of these risks has increased in the US, in-depth understanding remains limited. In Canada, awareness is higher but has remained stagnant over the past decade. In the UK, most people recognize the adverse health effects of climate change; although, there is no comparable evidence on the understanding of specific health impacts or changes in awareness over time. Beyond these countries, there may be considerable awareness of the health impacts of climate change, with some people in particularly high-risk regions possessing a deep understanding of their increased risks. However, accurately gauging the extent of this knowledge is challenging due to many studies outside the US, UK, and Canada relying on small, unrepresentative samples.

## How People Respond to Information About the Health Implications of Climate Change

### Effectiveness of Framing Climate Change as a Health Issue

Framing climate change and air pollution as public health issues—rather than as environmental, national security, or economic issues—can be an effective way to engage the public (Myers et al., 2012; Petrovic et al., 2014). Climate policies with a public health focus have been shown to garner significantly greater support than frames that solely emphasize climate-related benefits (Walker et al., 2017). A recent multinational study conducted across five countries—China, Germany, India, the UK, and the US—found that health and environmental frames garnered higher public support for climate policies compared to economic and migration frames. Additionally, presenting climate change as a global issue with immediate consequences also increased support for these policies (Dasandi et al., 2022).

While a health frame has shown potential for increasing public concern and support for climate solutions, some studies have identified limitations in its effectiveness (Bernauer & McGrath, 2016; Hart & Nisbet, 2012; Levine & Kline, 2017; McCright et al. 2016). McCright et al. found that the effectiveness of the health frame was not robust when Americans, especially conservatives, were also exposed to a climate change denial counter-frame, highlighting the challenge of communicating effectively about the health relevance of climate change in the face of misinformation and denial.

Similarly, Levine and Klein, (2017) found that framing climate change as a risk to personal health can have a paradoxical effect among Americans; it increases their concern about climate change but reduces their willingness to engage in collective political action to address it (i.e., sign an online petition addressed to politicians to end fossil fuel extraction and move towards clean energy). The authors suggest that reminding people of their own health vulnerabilities may make them feel less capable of investing personal resources into political participation. To counteract this effect, it may be important to emphasize the collective health risks of climate change (vs. the personal health risks) and boost people’s sense of response efficacy by focusing on the health benefits to be gained from climate policy as opposed to the health harms avoided (Dasandi et al., 2022; Levine and Kline, 2017).

Research conducted in the US suggests messages highlighting the negative health impacts of fossil fuels and air pollution have been found to increase public understanding of the issue, support for clean energy, and willingness to engage in advocacy for solutions (Feldman & Hart, 2018; Hart & Feldman, 2021; Kotcher et al., 2019b, 2021). In particular, one study found that justifying clean energy policies in terms of their contribution to reducing air pollution as opposed to climate change is more likely to gain the support of American conservatives (i.e., Republicans; Hart & Feldman, 2018).

### Impact of Informing About Health Risks of Climate Change & Air Pollution

A growing body of research, primarily conducted in the U.S., suggests that informing people about the health impacts of climate change and fossil fuels can increase their concern and engagement with these issues (Kim et al., 2021; Kotcher et al., 2018, 2019b, 2021; Kreslake et al., 2016). Additionally, information about climate change’s health risks increased people’s intentions to contact their elected officials to advocate for climate and health solutions (Kotcher et al., 2021). Emphasizing the climate-related health risks of mosquito-borne diseases such as dengue fever also increased support for climate change mitigation policies among climate change skeptics (Motta et al., 2023).

Research provides evidence-based strategies for effectively communicating the health risks of climate change (Adebayo et al., 2020; Hart & Nisbet, 2012; Kreslake et al., 2016; Limaye & Toff, 2023). Adebayo et al. (2020) found that narrative-based information about the health risks of climate change was more effective than didactic information in increasing pregnant women’s knowledge, risk perceptions, self-efficacy, intentions to adopt risk-reducing behaviors, and subsequent information-seeking behavior. Kreslake et al. (2016) suggest that providing climate and health information to vulnerable populations likely increases their knowledge, certainty that climate affects their health, and intentions to change their behavior to reduce risks. Limaye and Toff (2023) found that informing people about the healthcare costs associated with climate change can increase perceptions of climate change risk, especially when communicated on a per-household level rather than a national scale.

Visual imagery is another strategy to communicate climate change and health, although there is limited evidence on best practices for using such strategies and their effectiveness. Fear-inducing images linked to climate impacts effectively capture attention but can elicit strong negative emotions (Leviston et al., 2014; Chapman et al., 2016) and lower self-efficacy, making participants feel less capable of addressing climate change (O’Neill et al., 2013; O’Neill & Nicholson-Cole, 2009). Conversely, images showcasing climate solutions have been associated with increased perceived self-efficacy but reduced issue saliency (O’Neill et al., 2013; Metag et al., 2016). However, some research contradicts this apparent trade-off. A study conducted in Germany, the UK, and the US found that while climate-impact images generated negative emotions, they had a positive influence on various outcomes, including behavior change, sharing of the image, comprehension, and motivation to seek more information (Chapman et al., 2016). Moreover, images featuring ‘real’ people, rather than staged or clichéd depictions like politicians or protestors, were perceived as more credible and authentic, eliciting greater concern and motivation to act.

The only study of public perception of climate and health-specific imagery, a survey in the United Kingdom, found that images depicting air pollution were most effective in generating concern and increasing self-efficacy. Approximately 75% of respondents believed they could personally address air pollution as a climate impact, compared to only 12% for heat stress, 6% for flooding, and 7% for infectious diseases (Climate Outreach, 2020).

### Impact of Informing About Health Benefits of Climate Solutions

Limited research, primarily conducted in Western countries, has focused on communicating the health benefits of climate solutions. However, the evidence collected thus far suggests it may be perceived by the public as clearer and more useful information than risk information.

Furthermore, communicating the health benefits of climate solutions can help build support for climate policies (Dasandi et al., 2022; Spence & Pigeon, 2010) and renewable energy policies (Stokes & Warshaw, 2017), mobilize people to engage in advocacy (Levine & Klein, 2019), and increase willingness to adopt individual mitigation actions (Amelung et al., 2019; Hermann et al., 2020). Presenting solutions-focused information may also help activate positive emotions, like hope, which have been found to be strongly associated with pro-climate intentions (Chadwick, 2015; Feldman & Hart, 2016; Nabi et al., 2018; Ojala, 2012).

One study found that Americans are most likely to respond to information that includes the risks posed by climate change to health, potential solutions, and a call-to-action (Kotcher et al., 2021). Of these three message categories, solutions information was the most effective, although the messaging that included all three was substantially more effective than messaging that included only one or two categories of information.

### Depolarizing Potential of Health Messaging

Focusing on the health risks of climate change and the health benefits of solutions can help make the issue less politically divisive (Dearing & Lapinski, 2020). Additionally, presenting climate change in a health context makes it more concrete, rendering it salient and relevant to the public.

Several studies have shown that health-framed messages can effectively increase support for climate mitigation policies across political lines. Notably, research conducted in the US, the UK, and China found that positive, health-framed messages were particularly effective even among conservatives or individuals who tend to be less concerned about climate change Dasandi et al., 2022). This finding aligns with results from other studies (Kim et al., 2021; Kotcher et al., 2018, 2021; Maibach et al., 2010; Myers et al., 2012). For instance, information about the health harms of climate change can increase worry and risk perception, particularly among those who identify as moderate and somewhat conservative (Kim et al., 2021; Kotcher et al., 2018). Additionally, Kotcher et al. (2019b) found that people across the political spectrum considered neurological health problems from air pollution in babies, young children, and older adults to be the most concerning health implications of air pollution from fossil fuels.

However, one study found a potentially important limitation. Hart and Nisbet, (2012) found that Republicans’ responses to stories about the health impacts of climate change varied depending on the perceived victims. When the health impacts were depicted as affecting socially distant individuals (i.e., those living outside the US), conservatives were less likely to support climate action than when the victims were depicted as close to home (Tables 1 and 2).

## Discussion & Conclusion

Our review suggests there is considerable value in communicating about climate change as a health issue. Emphasizing the health risks associated with climate change and highlighting the potential health benefits of climate solutions makes the topic more concrete and salient while minimizing its potential for political divisiveness. Specifically, information about the health harms of climate change and air pollution can be a powerful catalyst for increased support for clean energy transitions and informed action. Highlighting the health benefits of clean energy, reduced air pollution, and other climate actions can effectively build public support for pro-climate policies, mobilize action, and inspire hope. Additionally, visual images depicting ‘real’ people experiencing the health harms of climate change, the benefits of solutions, and engaging in climate actions make communication more authentic and relatable, eliciting greater concern and motivation from audiences.

Climate and health messaging, like all effective communication, is not a one-size-fits-all approach. Communicators must carefully conduct audience analysis to understand their target audience and be intentional about the intended effect. For instance, they need to determine whether the goal is to shift knowledge, change individual attitudes, influence behavior, or increase policy support.

### Knowledge Gaps & Future Directions

While the existing research provides valuable insights, the knowledge base remains incomplete. Most studies on public perceptions of climate change and health with large representative samples have been conducted in the Global North, leaving a significant knowledge gap in understanding how people outside these regions perceive climate change and health. Therefore, conducting comprehensive research to assess public understanding of climate change and health in diverse global populations is critical, particularly as many in the Global South are at increased risk of climate health impacts. This research should examine how cultural, social, and contextual factors shape the understanding of climate and health messages. To achieve this, funding should be directed towards building capacity by providing training, resources, and infrastructure to support locally-based researchers. Additionally, creating communities of practice will enable these researchers to share knowledge, collaborate effectively, and conduct high-quality research on this important topic.

Research on communicating the inequities of climate change’s disproportionate health impacts on vulnerable populations—such as low-income communities, ethnic and racial minorities, women, children, and those with chronic illnesses or outdoor jobs—is limited (Pearson et al., 2017). The field must make swift strides to develop and test communication strategies for diverse audiences to advance equity in climate and health messaging and promote climate justice and action.

This review demonstrates the urgent need to improve public understanding of the significant health risks posed by climate change and provides actionable insights and strategies for effectively addressing this critical issue.

**Table 2:** provides a summary of key findings on public understanding and communication of climate and health.

Theme	Key findings	Audience/region differences	Total number of studies on topic	Notable studies
Public Perceptions of Climate Change & Health	Awareness of climate-related health risks is increasing in the US, though in-depth understanding remains limited. In the UK, perceptions are shaped by personal experience and knowledge of specific impacts. In India, over half the population sees climate change as a driver of serious health risks. Widespread awareness of climate change as a health threat appears to exist across the Global South, but broader insights are limited due to small, unrepresentative samples	US: Awareness varies by climate attitudes, political ideology, age, and race Global South: Awareness is often rooted in direct experience with local climate impacts. Some African countries show a deep understanding of specific health consequences	49 studies — Asia (17), Africa (10), North America (9), Europe incl. UK (6), South America (4), Caribbean (1), Multinational (2; US, UK & Malta; Myanmar, Netherlands, Spain, UK & US)	Kotcher et al. (2020); Roser-Renouf et al. (2021); Harrison & Graham (2022); Casson et al. (2023); Leiserowitz et al. (2022); Akerlof et al. (2010); Bryson et al. (2021)
Effectiveness of Framing Climate Change as a Health Issue	Framing climate change as a health issue is more effective than economic, security, or environmental frames in boosting concern and policy support. Messaging on the health impacts of air pollution from fossil fuels is also impactful	Most effective among those typically unconcerned about climate change. However, effectiveness can be limited when audiences don’t relate to affected groups, or perceive low agency, especially when exposed to opposing narratives	15 studies — US (13), UK (1), Multinational (1; China, Germany, India, UK & US)	Myers et al. (2012); Dasandi et al. (2022); Levine & Kline (2017); Kotcher et al. (2021)
Impact of Informing About Health Risks of Climate Change	Sharing information on health risks increases concern, engagement, policy support, and advocacy intentions. Narrative format, personally relevant content, and visual imagery have shown to be particularly effective	Health-risk messaging resonates across the political spectrum, especially among moderates and conservatives, when they are concrete, personally relevant, and socially proximate. Neurological impacts on babies, young children, and older adults are viewed as the most concerning effects of fossil fuel-related air pollution	15 studies — US (9), UK (2), Australia (1), Multinational (3; US, UK & Germany; Germany, Switzerland & Australia; Australia, UK & US)	Kotcher et al. (2018, 2019b, 2021); Kim et al. (2020); Feldman & Hart (2018); O'Neil et al. (2013); Climate Outreach (2020)
Impact of Informing About Health Benefits of Climate Solutions	Communicating the health benefits of climate solutions can increase policy support, advocacy intentions, and personal action. However, messages that communicate risks, solutions, and calls to action are most effective	Health-benefit messaging can appeal across the political spectrum. However, more research is needed to understand variations across different groups and geographic contexts	12 studies — US (7), Sweden (1), Germany (1), UK (1), Multinational (2; China, Germany, India, UK & US; France, Germany, Norway & Sweden)	Chapman et al. (2016); Dasandi et al. (2021); Kotcher et al., (2021); Nabi et al. (2018)

## Supplementary Information

Below is the link to the electronic supplementary material.

Supplementary file1 (XLSX 84 kb)

## Data Availability

A full list of articles that met the inclusion criteria for this review and search strings for each language can be found in the Supplementary Material.

## Footnotes

1 A full report covering findings from public, health professional, and policymaker audiences is available online: Uppalapati, S., Ansah, P., Campbell, E., Gour, N., Thier, K., Kotcher, J., & Maibach, E. (2023). A global review of research on effective advocacy and communication strategies at the intersection of climate change and health. George Mason University. https://doi.org/10.31219/osf.io/6w3qh
